# Recent advances in polymeric chemically amplified positive resists: Materials and design strategies

**DOI:** 10.1002/smo2.70077

**Published:** 2026-07-28

**Authors:** Litao Zhou, Haoyuan Li, Jianhua Zhang, Hanshen Xin

**Affiliations:** ^1^ School of Microelectronics Shanghai University Shanghai China; ^2^ Department of Chemistry College of Sciences Shanghai University Shanghai China

**Keywords:** additives, chemically amplified resists, functional units, photoacid generators, resin backbones, sensitization strategies

## Abstract

Chemically amplified resists (CARs) are essential for advanced photolithography but face critical challenges at extreme ultraviolet (EUV) nodes, including the resolution–line‐edge roughness–sensitivity (RLS) trade‐off and low photon utilization. To provide theoretical guidance for the design of next‐generation CARs, this review systematically discusses the molecular design of positive CARs, focusing on photoacid generators (PAGs), polymer architectures, functional monomers, and additives. We discuss PAG evolution from classical onium salts to polymer‐bound and naphthalimide‐based systems with tunable structure–property relationships, alongside polymer backbone strategies including styrenic, acrylate, norbornene, and self‐immolative platforms. The roles of functional monomers and additives are also discussed. Emerging strategies to sensitize resists—self‐amplification, controlled polymerization, copolymerization of sensitive monomers and photosensitized CARs—are comprehensively reviewed. Finally, current challenges, along with design strategies for each component of the photoresist, are summarized. This review provides a unified framework connecting molecular design to lithographic performance, serving as a resource for researchers and a roadmap for next‐generation CAR development.

## Graphical abstract

This review summarizes recent advances in the molecular design of positive chemically amplified resists, focusing on photoacid generators and polymer architectures. Key strategies for enhancing sensitivity—including self‐amplification effects, copolymerization of sensitive monomers, controlled polymerization, and photosensitized chemically amplified resists—are systematically discussed. The fundamental RLS trade‐off and future directions for advanced lithography are also highlighted.

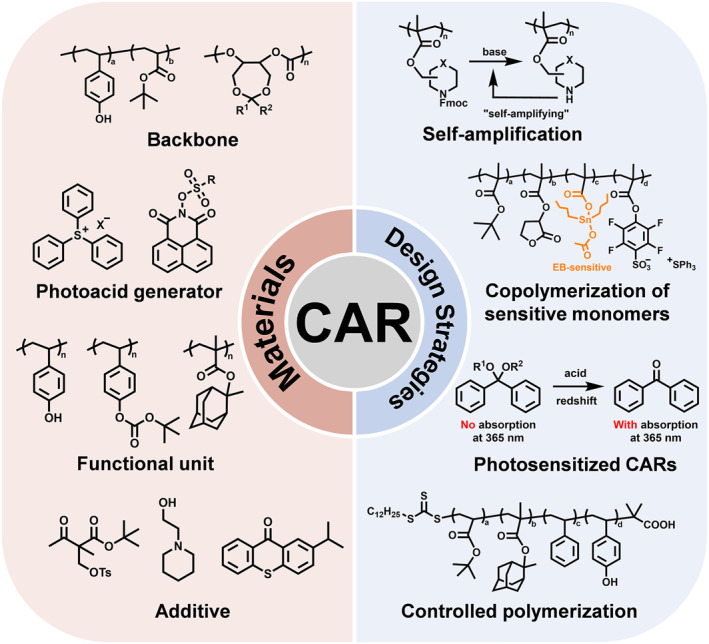

## 1 INTRODUCTION

Photolithography stands as one of the most transformative technologies of the modern era, enabling the fabrication of integrated circuits that power virtually every aspect of contemporary life.^[1]^ Among the various classes of photoresists developed over the decades, chemically amplified photoresists (CARs) have emerged as the dominant platform for advanced lithography, revolutionizing semiconductor manufacturing through their unique signal amplification mechanism.^[2]^

**FIGURE 1:**
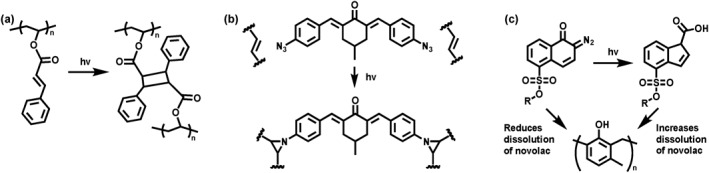
Early photoresist structures: (a) Cinnamate‐based cycloaddition cross‐linking system; (b) Azide‐rubber cross‐linking system; (c) DNQ‐Novolac system.

The semiconductor era demanded more robust materials. Louis Minsk developed poly(vinyl cinnamate), synthesized via the post‐polymerization esterification of poly(vinyl alcohol) with cinnamoyl chloride.^[3]^ This material exhibited excellent shelf‐life and enhanced etch resistance, with photo‐induced [2 + 2] cycloaddition of cinnamate moieties resulting in cross‐linking reactions (Figure 1a). However, poor adhesion to SiO_2_ substrates hindered its practical application. To address this adhesion issue, Kodak invented the Kodak thin film resist (KTFR) by combining photoactive bis‐aryl azide with pretreated synthetic rubber (Figure 1b).^[4]^ Despite its success as a negative‐tone resist, KTFR suffered from swelling during development with non‐polar solvents, limiting resolution to approximately 2 μm. The transition to positive‐tone resists marked a significant paradigm shift. The Novolac/diazonaphthoquinone (DNQ) system was developed, consisting of cresol Novolac polymer combined with photosensitive DNQ.^[5]^ In this two‐component formulation, DNQ acted as a dissolution inhibitor, making Novolac insoluble in an aqueous base. Upon UV exposure (i‐line, 365 nm), DNQ underwent Wolff rearrangement to form indenecarboxylic acid, which acted as a dissolution accelerator (Figure 1c). This system enabled the fabrication of early sub‐micron devices and remains in use today. However, a by‐product of this reaction was nitrogen gas, which was outgassed from the resist film—an issue that would persist as a concern for high‐resolution lithography. Early photoresist systems suffered from common limitations: low sensitivity and poor adaptability to different wavelengths. As the lithographic wavelength has shortened, the available optical source power has significantly decreased, thereby creating a pressing need for more sensitive photoresist systems.^[6]^

**FIGURE 2:**
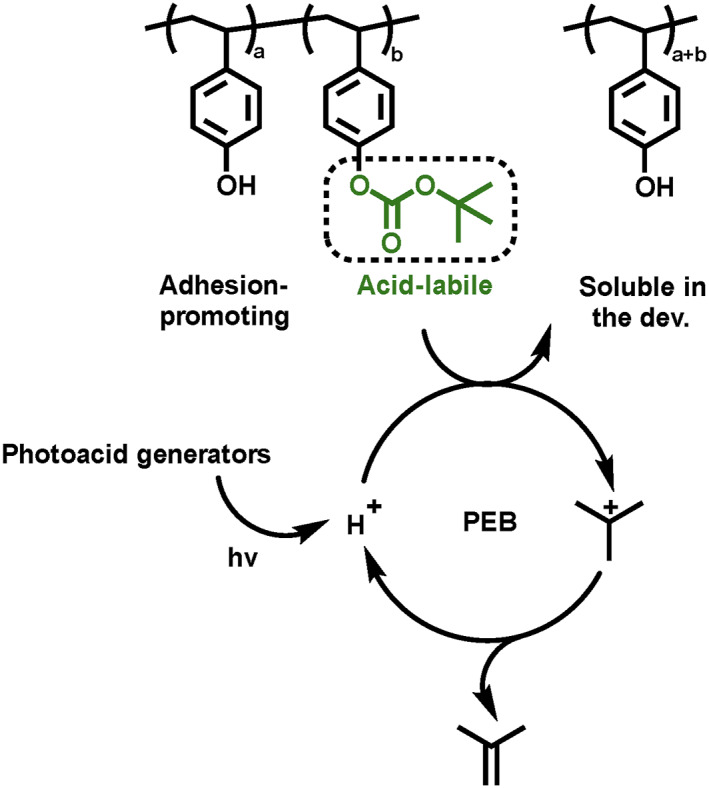
Schematic diagram of the mechanism of chemically amplified resists.

The invention of CARs in the early 1980s represented a key development in lithographic materials science. Willson, Fréchet, and Ito at IBM Almaden research center developed the first tert‐butoxycarbonyl(*t*‐Boc)‐protected *p*‐hydroxystyrene system CAR, solving the critical challenge of low sensitivity at 248 nm where mercury light sources produced very little UV light.^[7]^ The fundamental mechanism of CARs involves several steps as shown in Figure 2: (1) exposure generates acid from a photoacid generator (PAG); (2) post‐exposure bake (PEB) allows the acid to catalyze deprotection of acid‐labile groups on the polymer; (3) the regenerated proton continues to catalyze subsequent reactions, creating an amplification cascade; (4) development in aqueous base removes the deprotected (soluble) regions for positive‐tone resists.^[8]^ The key innovation was the concept of chemical amplification: a single photon‐generated acid molecule could catalyze multiple deprotection reactions, substantially enhancing sensitivity without sacrificing resolution.^[9]^ Owing to chemical amplification, the CARs exhibited excellent lithographic performance under low‐dose irradiation.

The development of CARs faces the fundamental RLS (resolution, line‐edge roughness [LER], sensitivity) trade‐off: resolution, LER, and sensitivity cannot be simultaneously optimized. Acid diffusion during PEB blurs latent images, limiting resolution. At advanced nodes (EUV), stochastic effects—photon shot noise and non‐uniform acid distribution—cause critical defects. Sensitivity directly determines lithography throughput, as higher sensitivity allows lower exposure doses and faster wafer processing—a critical factor for cost‐effective manufacturing, and higher sensitivity permits the use of a lower exposure dose, which in turn reduces stochastic effects. Moreover, sensitivity is central to the fundamental RLS trade‐off, making it essential for achieving defect‐free patterning at advanced technology nodes.^[10]^

**FIGURE 3:**
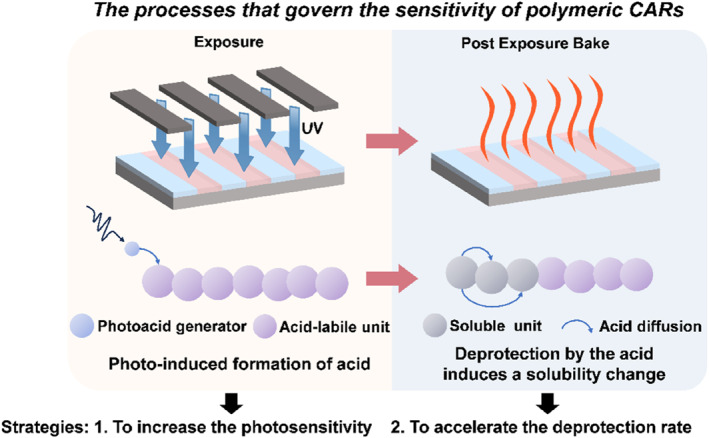
Mechanisms governing photoresist sensitivity and related enhancement strategies.

Based on the above, two primary strategies have been pursued to enhance the sensitivity of photoresists. One objective is to enhance the photosensitivity of the resist while the other is to accelerate the degradation rate of the polymer resin (Figure 3). For PAGs, the development direction is to strengthen the study of structure‐property relationships to achieve: (1) high absorption; (2) high acid generation efficiency; (3) low acid diffusion; and (4) minimal side reactions. As for photoresist resins: (1) controllable monomer sequence within the polymer chain and lower polydispersity; (2) protecting groups with high sensitivity to acids/bases; (3) various novel sensitization strategies—including the relationship between main‐chain scission/self‐immolation/self‐amplification effects and photoresist performance; and (4) high etch resistance/selectivity. To this end, significant research efforts have been dedicated to improving both the sensitivity and overall lithographic performance of photoresists.

While numerous reviews have been written on the performance of CARs,[^7^, ^10^, ^11^, ^12^] few have discussed the molecular‐level design strategies of their various components. To provide theoretical guidance for the design of next‐generation CARs, this review provides a comprehensive overview of the recent advances in polymeric CARs, structured according to their key components. We will systematically discuss the developments in PAGs, resin backbones, functional units, and additives. Furthermore, we will delve into critical concepts, including the application of the self‐amplifying effect in CARs, copolymerization of sensitive monomers, the impact of controlled/living polymerization on resin properties, the influence of polymer sequence, and photosensitive CARs system on overall lithographic performance. In parallel, this review will also highlight our own laboratory's research philosophy on the design of advanced photoresists and conclude with our perspectives on the future trajectory of CAR development.

## 2 PHOTOACID GENERATOR OF CARS

The PAG is indispensable in CARs, as it initiates the catalytic amplification cascade that enables high sensitivity at low exposure doses. The sensitivity of a CAR is determined by multiple factors, including the absorption wavelength and absorption efficiency of the PAG, its acid‐generation efficiency, as well as the strength and diffusion behavior of the generated acid in the resist matrix.^[13]^ First of all, PAG must exhibit strong absorption at the exposure wavelength—whether 365 nm, 248 nm, 193 nm for i‐line and conventional DUV systems. High absorption efficiency ensures that incident photons are effectively utilized, while high acid generation efficiency (quantum yield) maximizes the number of acid molecules produced per absorbed photon.^[14]^ In addition, both the acidity of the photogenerated acid and its diffusion behavior are important factors that influence the resist performance. The performance and design strategy of the same photoacid can differ under different lithographic techniques. Under i‐line and DUV exposure, PAG design is more directly related to optical absorption characteristics and photolysis efficiency at the corresponding wavelength, whereas under EUV and electron beam lithography (EBL) exposure, PAG decomposition is more strongly influenced by secondary electrons, low‐energy electron capture, and subsequent dissociative processes.^[15]^

PAGs can be broadly classified into ionic and non‐ionic categories, each offering distinct advantages for photopolymer applications. Ionic PAGs, predominantly comprising diaryliodonium and triarylsulfonium salts with anions such as PF_6_^−^, SbF_6_^−^, and AsF_6_^−^, exhibit excellent thermal stability and high acid generation efficiency. These onium salts operate primarily through heterolytic or homolytic cleavage upon UV excitation, generating strong Brønsted acids. However, their intrinsic absorption is limited to wavelengths below 300 nm, making them inherently suitable for i‐line (365 nm) incompatible applications unless structurally modified.^[16]^ Their practical application is often limited by their poor solubility. In contrast, non‐ionic PAGs exhibit good solubility but typically suffer from inferior thermal stability and lower acid generation efficiency. Consequently, the chemical modification of PAGs has remained a major focus of research in the field of photoresists, aiming to balance these competing properties.^[13]^

Diazonaphthoquinone (DNQ) sulfonates (**1**), as the most classic non‐ionic PAGs, have been extensively employed in novolac‐based photoresists.^[5]^ In contrast, the application of other non‐ionic PAGs, such as 2‐nitrobenzyl sulfonates (**2**), has been limited by issues like undesirable free radical side reactions during the lithographic process.^[16]^ Irgacure 103 (**3**), a commercial i‐line PAG, shows mediocre lithographic performance due to its weak photogenerated acid.

Nonionic PAG such as phenolic (**4**), oxime (**5**), naphthalimide oxime (**6**), and maleimide oxime esters (**7**), due to its low acid‐generation efficiency, this system has received limited research attention.^[13]^ Introducing halogens into nonionic photoacid generators is a common modification strategy. Ober and co‐workers reported that modified maleimide oxime esters (with iodine introduced on the sulfonic acid side) significantly enhance the sensitivity in EUV lithography, achieving a sensitivity of 1.4 mJ/cm^2^.^[17]^ Conversely, ionic PAGs continue to serve as the dominant choice in practical applications. Triphenylsulfonium (TPS) sulfonates (**8**) are the most commonly used ionic PAGs. Consequently, the sulfonium cation has been the subject of extensive chemical modification by researchers. Kizu et al.^[18]^ developed selenonium‐based PAGs (**9**) for EUV lithography, achieving significantly improved sensitivity (*E*_0_ = 0.6–0.8 mJ/cm^2^) compared to conventional sulfonium PAGs due to the high EUV absorption cross‐section of selenium. Rohm & Haas Electronic Materials disclose novel telluronium‐based photoacid generators (**10**) that exhibit enhanced EUV absorption compared to conventional sulfonium counterparts, thereby offering improved performance for extreme ultraviolet lithography.^[19]^

Wang's group developed polymeric sulfonium salt PAGs including poly(1‐(benzoyl‐methyl)‐tetrahydrothiophenium *p*‐styrenesulfonate‐co‐tertiary‐butyl methacrylate) (polymeric PAG) **(11)** and related copolymers, achieving an exposure dose of 70 mJ/cm^2^ for 0.35 μm resolution in thick‐film 248‐nm photoresists and 30 mJ/cm^2^ for 0.175 μm resolution in one‐component KrF resists.[^20^, ^21^] Iwasa et al.^[22]^ developed thermally stable alkylsulfonium salts including 2‐oxobutylthiacyclopentanium trifluoromethanesulfonate (TTS5‐CF_3_) and 2‐oxobutylthiacyclopentanium nonafluorobutanesulfonate (TTS5‐C_4_F_9_), with TTS5‐CF_3_ showing high sensitivity of 7.8 mJ/cm^2^ at 150°C PEB and 16.1 mJ/cm^2^ at 120°C PEB for ArF lithography. Cyclopropyl‐containing sulfonium salt PAGs **(12)** such as cyclopropyldiphenylsulfonium triflate (CpDPSTf) designed by Kim,^[23]^ which demonstrated an exposure dose of 10 mJ/cm^2^ for 120–130 nm patterning with improved resolution compared to conventional triphenylsulfonium triflate. Additionally, they developed di(4‐t‐butylphenyl)iodonium perfluorobutanesulfonate (DTBPIPFBS) **(13)** for ArF CARs, achieving 15 mJ/cm^2^ sensitivity and 0.24 μm resolution at 44 mJ/cm^2^.[^24^, ^25^]

Perfluoro‐ and polyfluoroalkyl sulfonate PAGs have played an important role in the development of high‐performance CARs. Despite increasing regulatory restrictions on perfluoro‐ and polyfluoroalkyl sulfonate PAGs because of environmental concerns, these materials have historically been among the most important PAG classes in CARs.[^26^, ^27^] Their continued interest arises from the fact that fluorinated alkyl sulfonate anions can generate strong acids upon photolysis while the fluorinated alkyl chains also impart pronounced hydrophobicity. These characteristics can strongly influence acid strength, acid diffusion behavior, and resist compatibility, and thus play an important role in determining lithographic performance, including sensitivity, resolution, and LER. For this reason, perfluoro‐ and polyfluoroalkyl sulfonate PAGs have long been regarded as the key materials for achieving high‐resolution and high‐sensitivity patterning in CAR systems. At the same time, growing environmental and regulatory pressure has driven the development of alternative PAG chemistries with reduced fluorine content or non‐fluorinated structures, in an effort to maintain favorable lithographic performance while improving sustainability.^[28]^

**FIGURE 4:**
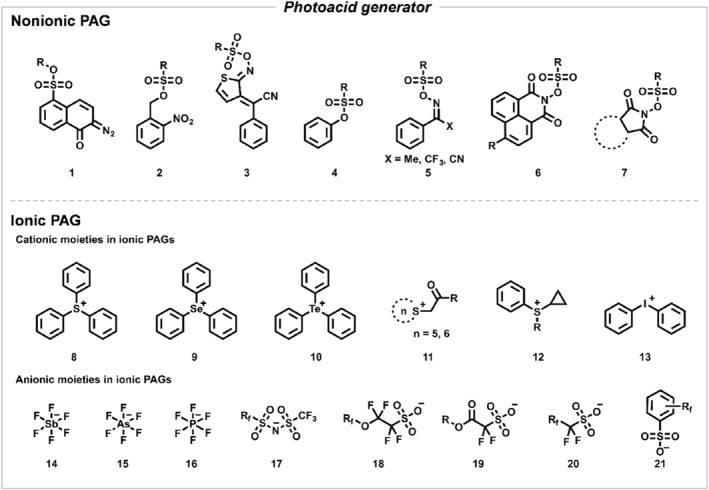
Common photoacid generators in chemically amplified resists.

Ito et al.^[29]^ reported that onium salts with different anions, including hexafluoroantimonate (**14**), hexafluoroarsenate (**15**), and hexafluorophosphate (**16**), as photoacid generators exhibit distinct lithographic performance in a three‐component resist system, where triphenylsulfonium hexafluoroantimonate achieves full development at an ultrahigh sensitivity of 2 mJ/cm^2^ (254 nm) with high contrast (*γ* = 4.2) and sub‐micron resolution, whereas other onium salts require higher exposure doses (15–59 mJ/cm^2^ at 313–365 nm), demonstrating that the anion structure of the photoacid generator critically determines acid strength, catalytic efficiency, and thus overall photospeed and imaging performance. The Gao group developed novel ionic PAGs with aromatic sulfonylimide anions (**17**) and a PAG‐bound copolymer via RAFT polymerization, achieving reduced acid diffusion length (∼50 nm) and improved line sidewall roughness (0.050 μm) (Figure 4).^[30]^

AZ Electronic Materials found a new class of nonionic photoacid generators with an ether‐containing fluorinated sulfonate anion (**18**); when formulated into 193 nm photoresists, the best formulation achieved a sensitivity of 30 mJ/cm^2^ for 70 nm dense lines and line‐width roughness of 13 nm.^[31]^ In Sumitomo Chemical's patent, using an ArF excimer laser as the exposure source, the photoacid generator containing a 1,1‐difluoro‐2‐oxoethanesulfonate (**19**) structure enabled first‐layer patterning at 43 mJ/cm^2^ and second‐layer patterning at 33–38 mJ/cm^2^, producing 100 and 150 nm line‐and‐space patterns.^[32]^

Clariant demonstrated that using sulfonium or iodonium salts of nonafluorobutane sulfonate or hexafluoropropane sulfonate (**20**) as photoacid generators in hydroxystyrene‐based CARs achieves sensitivity comparable to triflate‐based systems (18–24 mJ/cm^2^) and delivers dense line resolutions of 0.17–0.18 μm, demonstrating that larger, less volatile fluorinated alkanesulfonate anions effectively suppress outgassing and T‐top formation without compromising lithographic performance.^[33]^

Wang et al.^[34]^ reported that the acid generation efficiency of anionic photoacid generators (PAGs) bound to polymer resists follows the order: the PAG containing a perfluoroalkanesulfonate group with fluorine atoms directly attached to the carbon adjacent to the sulfonate (**20**, 150 mJ/cm^2^ at 254 nm) > the PAG with a nitro substituent on the benzene ring > the PAG with a tetrafluorobenzene ring (**21**) > the PAG with a trifluoromethyl substituent on the benzene ring (**21**). These results indicate that the sensitivity of fluorinated photoacids has been significantly improved.

**FIGURE 5:**
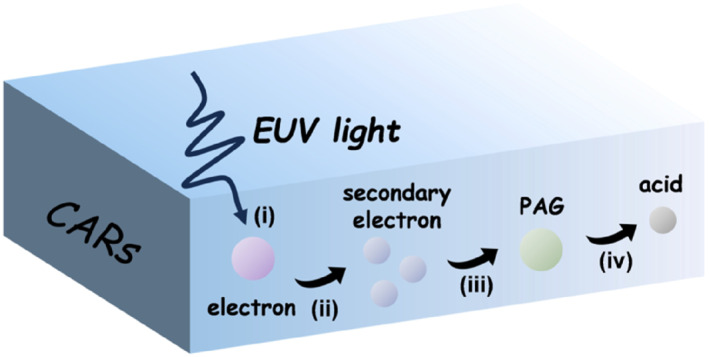
Schematic representation of radiation interactions between EUV photons and the CAR: (i) photon absorption and ionization; (ii) secondary electrons generation; (iii) electron reaction with PAG; (iv) acid generation for catalyzing deprotection.

Unlike DUV exposure, the chemistry of CARs under EUV irradiation is governed by ionization‐driven radiochemistry. A 13.5 nm EUV photon carries an energy of about 92 eV, which is sufficient to ionize resist components and generate primary photoelectrons. These photoelectrons subsequently undergo inelastic scattering and produce cascades of low‐energy secondary electrons (SEs), which are widely recognized as key reactive species in PAG decomposition and acid generation (Figure 5).^[35]^ Because of this electron‐mediated pathway, the lithographic response of CARs under EUV depends not only on the absorption cross‐section of the material but also on electron generation, transport, and energy dissipation. The finite migration distance of these electrons also introduces an electron‐blur effect, making structure–property relationships established under DUV exposure not directly transferable to EUV systems.^[36]^

This distinction is important when discussing molecular strategies for improving EUV sensitivity. The superior performance of high‐absorption PAGs, such as selenonium and telluronium‐based systems, is determined not only by their enhanced EUV absorption but also by their efficiency in electron‐induced decomposition. Likewise, the incorporation of sensitive or high‐EUV‐absorption monomers may affect not only absorption but also local energy deposition, SE‐mediated reactions, and stochastic patterning behavior. Therefore, rational molecular design for EUV resists requires consideration of both absorption properties and electron‐driven reactivity.

For PAG design, this means that maximizing photon absorption alone is insufficient. Under EUV exposure, PAG decomposition is widely considered to be strongly influenced by low‐energy electron capture and subsequent dissociative processes. Therefore, effective EUV PAGs should also possess favorable electron affinity to promote electron‐induced decomposition. Onium salts, such as sulfonium and iodonium salts, are attractive candidates in this regard because their electron affinity can be tuned through molecular design. In particular, incorporation of electron‐withdrawing substituents into the cation structure can lower the LUMO energy level, enhance electron capture, and facilitate reaction with low‐energy electrons in the resist film.^[37]^

Although the aforementioned PAG systems each possess distinct advantages, the RLS trade‐off remains a formidable challenge, polymer‐bound photoacid generators (PB‐PAGs) have attracted considerable attention as an effective strategy for improving the resolution performance of CARs. By covalently tethering PAG moieties to the side chain, the mobility of the acid source and, in some cases, the generated acid can be spatially constrained, which helps suppress acid diffusion, improve reaction localization, and reduce blur‐related effects such as LER. This feature is particularly attractive for high‐resolution lithography, where precise confinement of chemical reactions is essential.^[38]^

At the same time, PB‐PAGs also present several limitations. Their synthesis and purification are generally more complex than those of blended small‐molecule PAG systems, and the achievable PAG loading may be restricted by polymer architecture and processability requirements.^[34]^ In addition, incorporation of PAG units into the polymer may alter important resist properties such as glass transition temperature, solubility, film‐forming behavior, and dissolution characteristics. Moreover, while reduced acid diffusion can improve resolution, overly restricted acid transport may in some cases compromise efficient catalytic amplification and sensitivity. Therefore, the PB‐PAG design requires a careful balance among sensitivity, resolution, roughness, and process compatibility.

**FIGURE 6:**
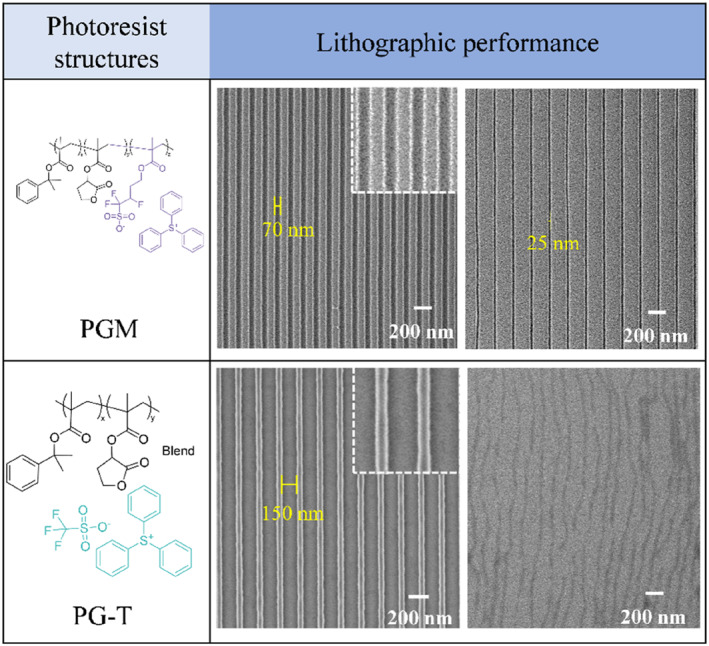
Enhancing photoresist performance using polymer‐bound photoacid generators. Reproduced with permission.^[39]^ Copyright 2024, Wiley Online Library.

To gain a deeper understanding of the respective advantages and disadvantages of polymer‐bound PAG and blended PAG strategies, our group conducted detailed studies on both approaches. We have compared photoresists formulated with blended and polymer‐bound PAGs, and developed an anionic PAG‐bound polymer photoresist (PGM) comprising 2‐Phenyl‐2‐propyl methacrylate (PPMA), γ‐butyrolactone methacrylate (GBLMA), and triphenyl sulfonium salt 1,1,2‐trifluorobutanesulfonate methacrylate (MTFBS‐TPS) (Figure 6). Compared to conventional PAG‐blend resists, our PGM system achieved superior sensitivity (27.9 μC/cm^2^), higher contrast (6.53), and improved resolution down to 25 nm in EBL and 37.5 nm in EUVL, attributed to reduced acid diffusion through covalent PAG attachment.^[39]^

**FIGURE 7:**
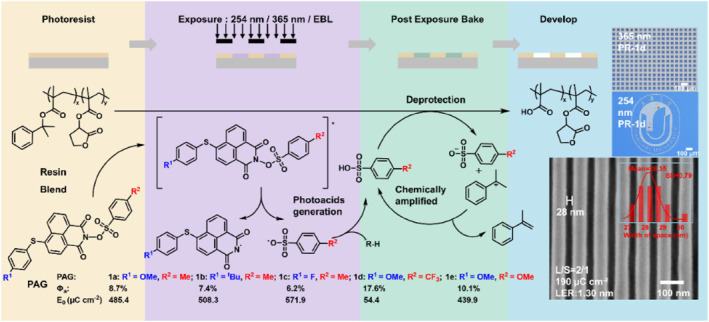
Structure‐property relationship study of naphthalimide‐based photoacid generators for enhanced photoresist performance. Reproduced with permission.^[14]^ Copyright 2025, Royal Society of Chemistry.

Motivated by two key limitations of unmodified naphthalimide PAGs—their narrow operational wavelength range and the general lack of systematic structure‐property relationship studies,^[40]^ we designed and synthesized five novel naphthalimide‐based nonionic sulfonate PAGs with tunable donor–π–acceptor configurations (Figure 7).^[14]^ By systematically adjusting the electron‐donating/withdrawing substituents on the naphthalimide and benzenesulfonate units, we established clear structure–property relationships, achieving acid generation quantum yields (*Φ*_a_) ranging from 6.2% to 17.6%. Notably, trifluoromethyl‐substituted PAG with optimal D–π–A structure exhibited the highest *Φ*_a_ (17.6%) and exceptional lithographic performance, including the highest sensitivity (*E*_0_ = 54.4 μC/cm^2^) and 28 nm resolution with low LER (1.3 nm) under EBL, demonstrating significant potential for advanced semiconductor manufacturing.

## 3 POLYMER BACKBONE OF CARS RESIN

The resin backbone serves as the structural foundation of the photoresist, providing mechanical integrity and determining the material's thermal stability, etch resistance, and solubility characteristics. Additionally, the backbone architecture—whether linear, branched, or containing cyclic units—directly influences acid diffusion behavior and the overall lithographic resolution.^[41]^

Styrene‐based polymers (**22**, **23**) are the most common backbone type in CARs.^[42]^ However, as lithography processes advanced to the 193 nm node, the strong absorption of their inherent benzene ring structure at this wavelength rendered them unsuitable for the application. Consequently, a series of non‐aromatic polymer backbones were developed, including radical copolymers of maleic anhydride and norbornene (**24**),[^43^, ^44^, ^45^] norbornene polymers prepared via ring‐opening metathesis polymerization (ROMP) (**25**),^[46]^ and rearrangeable acrylates (**26**, **27**).[^47^, ^48^] Photoresist resins featuring cyclic structures within their polymer backbone exhibit a significant advantage in terms of etch resistance.

Ueda^[49]^ and Kakimoto^[50]^ independently developed chemically amplified photosensitive polybenzoxazole systems using the same anthracene‐based PAG (DIAS), but with fundamentally different polymer architectures that led to distinct performance characteristics. Ueda employed a linear poly(*o*‐hydroxyamide) backbone (**28**) with norbornene end‐capping groups, achieving superior sensitivity of 60 mJ/cm^2^ and resolution down to 10 μm line/space patterns, attributed to the controlled molecular weight and cross‐linking capability of the terminal norbornene moieties that enabled high thermal stability up to 350°C. In contrast, Kakimoto utilized a hyperbranched poly(*o*‐hydroxyamide) scaffold (**29**) constructed from AB_2_‐type monomer self‐polycondensation, which offered enhanced solubility in common organic solvents and low solution viscosity due to its globular non‐entangled structure, though with modest sensitivity of 115 mJ/cm^2^ and larger feature sizes of 30 μm; the hyperbranched architecture required careful optimization of *t*‐Boc protection degree to balance dissolution rate and substrate adhesion, with 39 mol% *t*‐Boc content proving optimal. The linear system's rigidity and end‐group functionality favored high‐resolution lithography and extreme thermal durability, while the hyperbranched system's highly branched and compact morphology prioritized processability and film‐forming properties at the expense of some photospeed and resolution.

Innovative use of classic materials is also frequently applied to the development of photoresist resins. Kang et al.^[51]^ synthesized a CAR by using a Novolac as the base polymer and protecting a portion of its phenolic hydroxyl groups with Boc anhydride. In a similar approach, they also utilized tannic acid as a core material, modifying it into another CAR.^[52]^ Furthermore, they investigated the potential of these materials for subsequent device fabrication. Recently, they developed a dual‐sensitive functional photoresist based on Novolac backbone (**30**) modified with both diazonaphthoquinone (DNQ) units and acid‐cleavable ethyl vinyl ether (EVE) and Boc protection groups.^[53]^ This hybrid design synergistically combines DNQ photolysis with acid‐catalyzed deprotection, achieving 150 mJ/cm^2^ sensitivity and 612 nm resolution. The high hydroxyl content and structural diversity of the Novolac backbone, arising from its hydroxymethylation and dehydration condensation synthesis, necessitated careful optimization of modification degrees to balance solubility switching and alkaline developer resistance.

The construction of new single‐component polymers based on novel polymer synthesis methodologies has been a research hotspot in the field of photoresists in recent years.^[54]^ Wu et al.^[55]^ developed a CO_2_‐sourced polycarbonate (**31**) photoresist (PPhCXC) featuring a polycarbonate mainchain with acid‐labile cyclic acetal sidechains, synthesized via metal‐free alternating copolymerization of CO_2_ and epoxides. This novel CO_2_‐PC backbone delivers exceptional performance with 1.9 mJ/cm^2^ sensitivity and 7.9 contrast, surpassing commercial KrF (HTK1062) and ArF (PBMA) resists. The unique architecture enables water development through acid‐catalyzed hydrolysis of acetal groups into hydrophilic diols, eliminating toxic TMAH developers. The rigid cyclic structure in the backbone provides high thermal stability (Tg 92°C) and 38% superior etching resistance compared to poly(tert‐butyl acrylate), while achieving 750 nm resolution with narrow polydispersity (1.26–1.37) critical for low LER.

Main‐chain scission is a key mechanism in photoresists, where radiation directly breaks the polymer backbone. This reduces the molecular weight, making exposed areas more soluble for pattern development. Traditional poly(methyl methacrylate) (PMMA) resists of the main‐chain scission type are characterized by high‐energy electron‐beam‐induced carbon‐carbon bond cleavage (requiring high dose) and the absence of acid diffusion (owing to their non‐chemically amplified nature, which affords high fidelity).^[56]^ This compromise has led to the exploration of chemically amplified versions of main‐chain scission resists.^[57]^

Sulfone‐containing compounds possess intrinsic photosensitivity, causing them to undergo polymer chain scission upon irradiation. This property makes polysulfones a well‐established class of materials for photoresist applications.[^58^, ^59^, ^60^] Poly(*t*‐Boc‐styrene sulfone) (**32**), as a novel copolymer backbone synthesized via free radical polymerization, demonstrates significant advantages in CARs. Compared to traditional polystyrene or poly(α‐methylstyrene) matrices, this polysulfone backbone simultaneously enhances both sensitivity and contrast in deep‐UV lithography. For instance, Tarascon et al.[^61^, ^62^, ^63^] reported that a 2:1 poly(*t*‐Boc‐styrene sulfone) formulation containing 15 wt% 2,6‐dinitrobenzyl tosylate achieved a sensitivity of 26 mJ/cm^2^, a contrast exceeding 20, and 0.5 μm resolution capability. Furthermore, this backbone is compatible with both onium salt and nitrobenzyl ester PAGs, and undergoes acid‐catalyzed deprotection to yield aqueous base‐soluble poly(hydroxystyrene sulfone), further optimizing the lithographic performance.

Ober and coworkers developed poly(aryl acetal) polymers (**33**), a modular backbone‐degradable platform synthesized via Suzuki polycondensation,^[64]^ featuring tunable acid sensitivity and rigid polyaromatic structures for etch resistance. Comparing the work of the two previous studies, this poly(aryl acetal) platform fragments into phenolic terphenyl products upon acid cleavage and prioritizes synthetic flexibility and process control, achieving 22 nm resolution with 5.7 nm LWR under EUV exposure.

Self‐immolative polymers are considered highly promising candidates for the development of high‐sensitivity photoresists, which typically undergo triggered end‐to‐end or backbone depolymerization after an initiating event.^[65]^ Kohl et al.^[66]^ investigate a dry‐develop photoresist based on poly(phthalaldehyde‐co‐propanal) (**34**), which features an acid‐labile acetal backbone that depolymerizes into volatile monomers upon photoacid‐catalyzed cleavage. Using a commercial PAG (Irgacure PAG 103) (**3**) activated at 375 nm, they demonstrate that incorporating trihexylamine as a base quencher significantly enhances resist contrast from 1.1 to 2.5 and improves pattern fidelity, though sensitivity decreases from 90 to 100 mJ/cm^2^ for formulations with 10 wt% PAG. Non‐volatile residue and liquid‐phase formation during thermal development remain challenges, particularly for larger features. To address this, gradient exposure and plasma post‐treatment are shown to effectively mitigate residue, enabling cleaner all‐dry pattern transfer.

**FIGURE 8:**
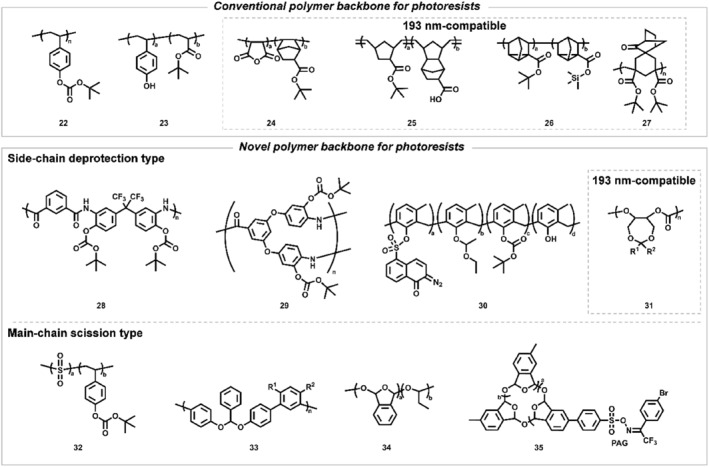
Backbone structure of chemically amplified resist resins.

PAG‐bound poly(phthalaldehyde) (PPA) (**35**) derivatives developed by Ober's group represent a significant evolution in self‐immolative resist design.^[67]^ Using sulfur(VI) fluoride exchange (SuFEx) click chemistry, they achieved modular synthesis of PPA with covalently bound non‐ionic PAGs, enabling the first single‐component resist with dual amplification: acid‐catalyzed depolymerization and PAG‐bound confinement. The PAG‐bound analogs exhibit considerably enhanced EUV sensitivity (3 mJ/cm^2^), higher contrast (*γ* up to 3.74), and no increase in surface roughness after development, and overcome the RLS trade‐off by tethering PAGs to the backbone, confining acid diffusion to reactive sites and improving both sensitivity and resolution (Figure 8).

## 4 FUNCTIONAL MONOMERS OF CARS RESIN

**FIGURE 9:**
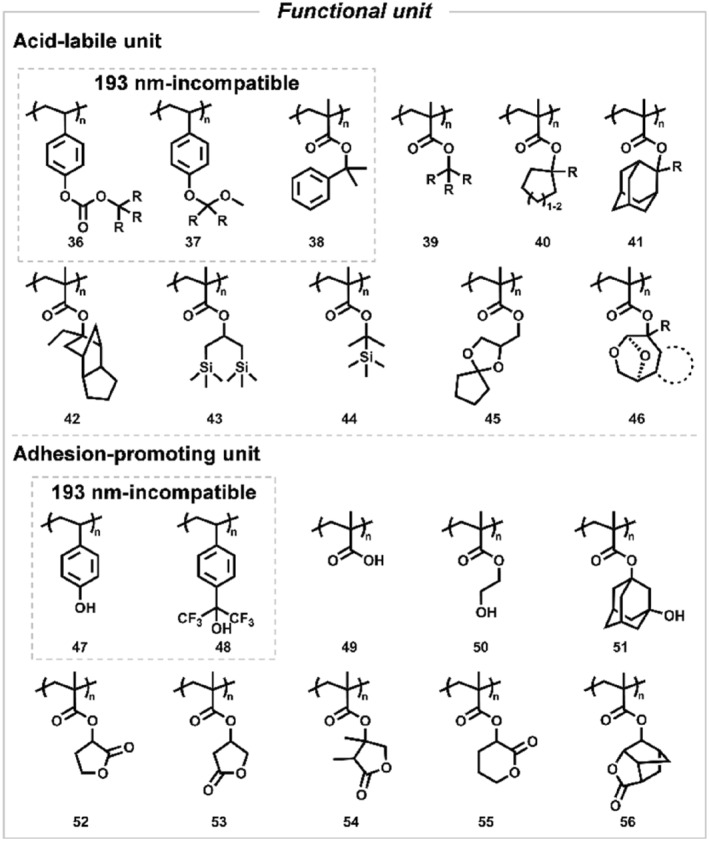
Typical functional moieties in photoresist resins.

Functional monomers in CARs can be primarily categorized into two types: acid‐labile monomers and adhesion‐promoting monomers. Acid‐labile monomers are crucial components in CARs, primarily used to control polymer solubility. These monomers, often containing *t*‐Boc^[65]^ or acetal groups,^[68]^ are grafted onto the polymer backbone (Figure 9). Upon exposure to photo‐generated acid and subsequent PEB, they undergo a deprotection reaction.^[2]^ This cleavage converts the polymer from hydrophobic to hydrophilic, rendering the exposed regions soluble in aqueous alkaline developer and enabling the formation of positive‐tone patterns.^[69]^

*t*‐Boc (**36**) is a foundational protecting group in CARs, whose acid‐catalyzed deprotection mechanism revolutionized the lithography industry by enabling high‐sensitivity patterning.^[70]^ Cameron et al.^[71]^ investigated three types of acid‐labile protecting groups in chemically amplified deep‐UV photoresists: acetals/ketals (**37**) with low activation energy (∼22 kcal/mol) for low‐temperature processing, *t*‐butyl carbonates (**38**) with medium activation energy (∼29 kcal/mol), and *t*‐butyl esters (**39**) with high activation energy (∼35 kcal/mol) for high‐temperature processing, where the low activation energy protecting groups enable faster deprotection and reduced bake temperature sensitivity while the high activation energy protecting groups provide better post‐exposure delay stability and profile shape through the annealing concept. Due to the presence of aromatic (phenyl) rings, protection group **36**, **37**, and **38** exhibit strong absorption at 193 nm, which renders them incompatible with lithography at this wavelength. This necessity prompted the development of non‐aromatic protecting groups (**40**–**46**), among which the adamantyl (**41**) and norbornyl (**42**) derivatives offer the additional advantage of high etch resistance owing to their rigid polycyclic frameworks. Additionally, Williams et al.^[72]^ developed methacrylic monomers and homopolymers derived from the sustainable building block dihydrolevoglucosenone (Cyrene, DLGO) with tertiary ester protecting groups, where poly(*n*‐BuMDLGO) (**46**) exhibited acid‐catalyzed deprotection at 130°C and achieved micrometer‐scale half‐pitch patterns using EBL with a sensitivity of 16.5 μC/cm^2^.

Adhesion‐promoting monomers are functional components incorporated into photoresist polymers to enhance interfacial bonding with the substrate, typically a silicon wafer.^[42]^ These monomers often feature polar functional groups, such as hydroxyl or carboxyl moieties, which increase the polymer's surface affinity and wettability.^[8]^ By improving the mechanical anchorage and chemical interaction at the resist‐substrate interface, they mitigate pattern collapse and prevent delamination during development, especially in high‐aspect‐ratio features. This adhesion enhancement is critical for maintaining pattern fidelity in advanced lithographic processes.

The phenolic hydroxyl groups in poly(*p*‐hydroxystyrene) (PHS) (**47**) facilitate strong interfacial interactions, typically through hydrogen bonding, thereby improving the adhesion of the photoresist film to various substrates.^[42]^ Driven by the same transparency requirements at 193 nm that motivated the development of aromatic‐free acid‐labile monomers, researchers also developed a series of non‐aromatic adhesion‐promoting monomers **49**–**56**.[^73^, ^74^, ^75^]

Fluorinated and hydroxyl‐functionalized monomers, commonly employed as high‐refractive‐index topcoats in 193 nm immersion lithography,^[6]^ are also finding novel applications in EBL. Kang and coworkers developed a novel fluorinated copolymer‐based CARs incorporating hexafluoro‐2‐hydroxypropyl styrene (HFS) (**48**) for high‐sensitivity lithography.^[76]^ The system achieves exceptional EB sensitivity (down to 7 μC/cm^2^) and 58 nm resolution through a dual amplification mechanism: enhanced absorption from fluorine content and C–F bond cleavage generating F^−^, which forms HF to catalyze additional deprotection.

## 5 ADDITIVE OF CARS

**FIGURE 10:**
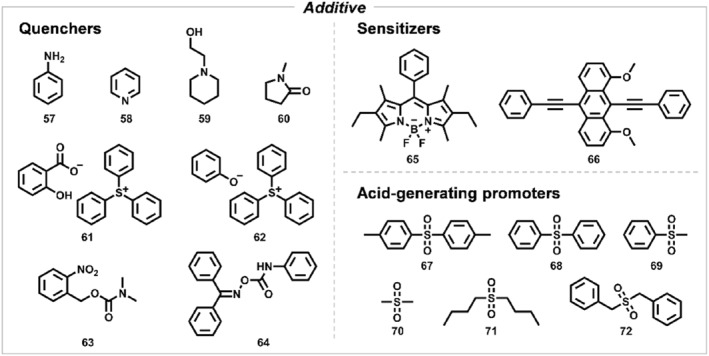
Common additives in chemically amplified resists.

In the formulation of modern CARs, the performance is finely tuned through the precise addition of several key components that work in concert with the polymer resin and the PAG. Among the most critical additives are quenchers, sensitizers, and acid‐generating promoters (Figure 10).

Quenchers (**57**–**64**), typically basic compounds like amines, are incorporated to neutralize stray acid and precisely control its diffusion during the PEB.[^77^, ^78^, ^79^] This control is essential for sharpening the latent image, preventing unwanted reactions in unexposed areas, and improving critical dimension (CD) control and overall process latitude. On the other hand, sensitizers (**65**,**66**) and acid‐generating promoters (**67**–**72**) are added to enhance the efficiency of acid production. A sensitizer works by absorbing light at the exposure wavelength—often more efficiently than the PAG itself—and, then transferring that energy to the PAG, triggering its decomposition.^[80]^ Similarly, an acid‐generating promoter is a substance that boosts the overall quantum yield of acid generation, ensuring that a sufficient concentration of acid is created to drive the catalytic deprotection reaction. The careful balance of these components is what allows for the high resolution, sensitivity, and stability required in advanced lithography.^[81]^

**FIGURE 11:**
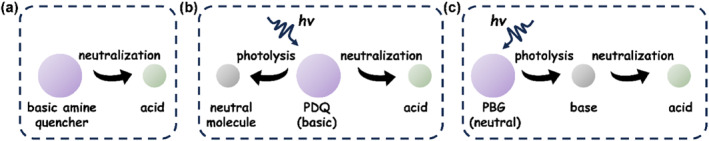
Mechanisms of different quenchers. (a) Basic amine quencher; (b) photo‐decomposable quencher; (c) photobase generator.

Quenchers are one of the key components in CAR formulations, together with the polymer and photoacid generator (PAG), because they influence acid diffusion, suppress background deprotection, improve image contrast, and balance the trade‐off between resolution, roughness, and sensitivity. Basic amine quenchers (**57–60**) (Figure 11a) are commonly used to neutralize part of the generated acid, thereby suppressing excessive acid diffusion and reducing unwanted background deprotection. This can improve resolution and LER, although excessive quencher loading may reduce sensitivity. Beyond conventional amine quenchers, more advanced systems such as photo‐decomposable quenchers (**61, 62**) (Figure 11b) and photobase generators (**63, 64**) (Figure 11c) have also been investigated. Photo‐decomposable quenchers lose their quenching ability upon irradiation, enabling a more dynamic control of the acid–base balance during exposure. In contrast, photobase generators release basic species upon photolysis, and therefore differ fundamentally from conventional quenchers in both function and timing.

The results obtained by Ku's group showed that photo‐base generators increase the local base concentration upon exposure, acting as a “brake” to neutralize excess acid, which is particularly beneficial for controlling iso‐dense bias in high‐density patterns. In contrast, photo‐decomposable quenchers undergo base degradation upon exposure, effectively reducing base concentration in irradiated areas and thus enhancing acid contrast and diffusion, making them suitable for high‐resolution patterning applications.^[82]^

**FIGURE 12:**
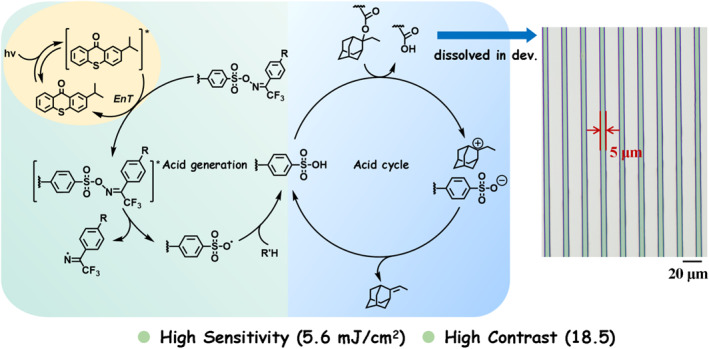
Enhancing 365 nm photoresist performance using 2‐isopropylthioxanthone as a photosensitizer. Reproduced with permission.^[87]^ Copyright 2025, ACS Publications.

The sensitization of oxime esters via a triplet‐triplet energy transfer (TTEnT) mechanism has attracted considerable interest in recent years, particularly within the field of synthetic methodology.[^83^, ^84^, ^85^, ^86^] In the meantime, oxime‐ester based PAGs have been widely adopted in advanced lithography in recent years.[^40^, ^67^] However, their inherent absorption window is relatively narrow, rendering them incompatible with i‐line exposure. In order to improve the acid generation efficiency and lithography wavelengths of non‐ionic PAGs, we recently used 2‐isopropylthioxanthone (2‐ITX) as a photosensitizer to enhance the performance of i‐line (365 nm) photoresists based on oxime‐ester PAGs (Figure 12).^[87]^ The PAG‐blended system achieved high sensitivity (*E*_0_ = 5.6 mJ/cm^2^) and high contrast (*γ* = 18.5). The results of this work also indicate that the substituents of the covalently bonded PAG can affect its relationship with the sensitizer — that is, the larger the steric hindrance, the poorer the performance of the sensitizer. This approach has enabled a broader applicable wavelength range for oxime ester‐based PAGs, leading to a substantial increase in the sensitivity of i‐line resists.

## 6 THE STRATEGIES FOR IMPROVING CAR PERFORMANCE

### 6.1 Self‐amplification effect for CARs

In conventional CARs, the catalytic chain length—the number of deprotection events catalyzed by a single acid molecule—typically ranges from a few hundred to several hundred cycles.^[88]^ In an effort to enhance the overall catalytic efficiency, researchers have designed and synthesized a series of molecules featuring a self‐amplifying effect to improve resist sensitivity. This effect, a form of autocatalysis, involves the generation of an additional acid or base molecule under the catalysis of the initial acid or base. This process, in turn, markedly accelerates the rate of the deprotection reaction. As a result, the photoresist can achieve an exceptionally high level of sensitivity.^[89]^

**FIGURE 13:**
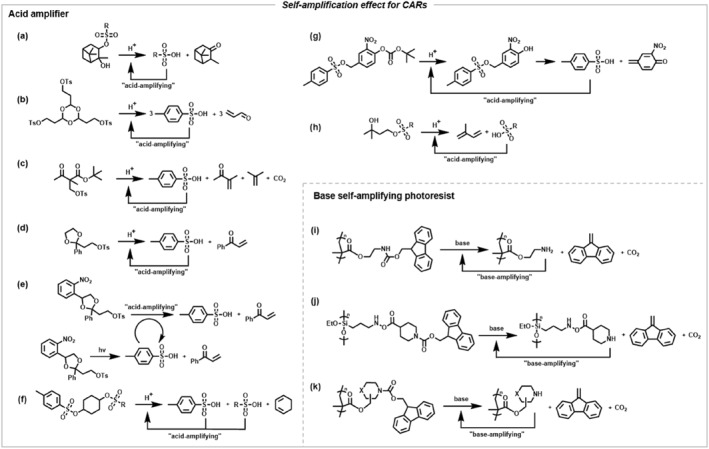
Application of a self‐amplifying effect in chemically amplified resists. (a–h) Acid self‐amplifying effect. (i–k) Base self‐amplifying effect.

The Ichimura group has developed a series of acid/base self‐amplifiers that play a pivotal role in enhancing photoresist sensitivity.^[90]^ Notable examples include the following: 1,2‐Diol monosulfonates (Figure 13a)^[91]^: undergo pinacol rearrangement to release sulfonic acid; pinanediol derivatives show half‐lives >500 min without acid but complete within minutes with acid, with gradients up to 390%/h. Trioxane (Figure 13b)^[92]^: generates three sulfonic acid molecules upon acid‐catalyzed fragmentation; stable without acid but shows sudden disappearance after the induction period with catalytic TsOH. Acetoacetates (Figure 13c)^[93]^: fragment via β‐elimination after acidolytic deprotection; tosylate and mesylate show sigmoidal conversion curves at 100°C, generating methyl isopropenyl ketone and sulfonic acid. β‐Ketals (Figure 13d)^[94]^: convert to β‐ketosulfonates then eliminate to generate sulfonic acid autocatalytically; exhibit sigmoidal curves for strong acid formation in solution. α‐Nitrophenyl‐substituted β‐ketals (Figure 13e)^[95]^: Enable phototriggered acid proliferation; UV exposure generates β‐ketosulfonate, which upon heating initiates autocatalytic TsOH generation without additional PAG. Cyclohexane‐1,4‐diol disulfonates (Figure 13f)^[96]^: Release two sulfonic acid molecules per unit via double β‐elimination; triflate derivative generates superacid autocatalytically. Benzyl sulfonates (Figure 13g)^[97]^: Undergo autocatalytic decomposition to release sulfonic acid; *p*‐hydroxybenzyl sulfonate with *t*‐Boc protection showed improved thermal stability, while the nitro‐substituted derivative displayed sigmoidal conversion with TsOH catalysis.

Brainard and coworkers designed 12 fluorinated acid amplifiers with tertiary or secondary triggers (Figure 13h).^[98]^ Tertiary AAs undergo autocatalytic decomposition (rate ratios 80–1400 at 100°C), while secondary AAs require stronger photoacids. Adding 70 mM 3MA to an environmentally stable chemically amplified photoresist (ESCAP) resist improved EUV performance: sensitivity ∼15 mJ/cm^2^, LER ∼5.0 nm at 50 nm features, and Z‐parameter improved threefold.

Compared to acid self‐amplifying photoresists, base self‐amplifying photoresists can avoid the issue of “air infection”, offer lower PEB temperatures, provide greater process latitude, and exhibit higher structural tunability; the research focus has evolved from additive‐based systems to self‐amplifying polymers, significantly enhancing the sensitivity and imaging performance. Ichimura et al.^[99]^ first proposed a polymer design with base‐amplifying units tethered to the side chains, solving the “air infection” problem caused by volatilization and excessive diffusion of small‐molecule base amplifiers. Upon photogenerated base triggering, the polymer underwent autocatalytic decomposition to yield polymer‐bound amino groups, achieving a sensitivity of 4 mJ/cm^2^ under 365 nm exposure and 130°C PEB, successfully producing micron‐scale positive patterns (Figure 13i).

Subsequently, Arimitsu et al.^[100]^ introduced base‐amplifying units into silicone resin backbones. By utilizing secondary amine generation with stronger basicity, the sensitivity was improved to 0.3 mJ/cm^2^ (254 nm exposure) (Figure 13j), an order of magnitude higher than the previous system, demonstrating the synergistic advantages of siloxane backbones in etch resistance and sensitivity.

**FIGURE 14:**
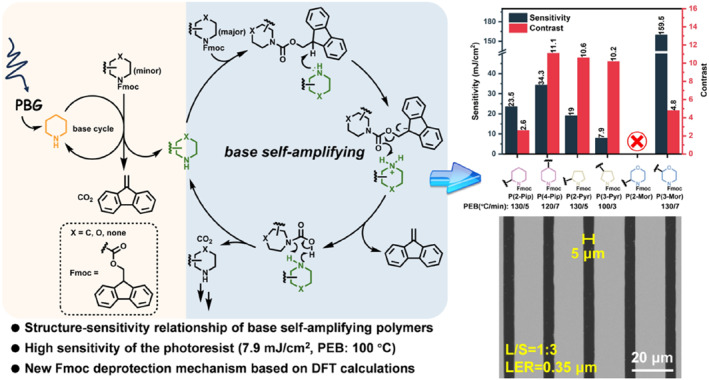
Improving the performance of photoresists by studying the structure‐sensitivity relationship of the base self‐amplifying polymers. Reproduced with permission.^[101]^ Copyright 2026, ACS Publications.

To address the lack of systematic structure‐sensitivity and theoretical studies in prior work, our team recently investigated the structure‐sensitivity relationships governing the base self‐amplifying effect, aiming to develop high‐performance photoresists. We investigated six polymers with different cores (piperidine, pyrrolidine, morpholine) and substitution positions through a combined approach of experimental work and density functional theory (DFT) calculations (Figure 14).^[101]^ P(3‐Pyrrolidine) exhibited the best performance: *E*_0_ = 7.9 mJ/cm^2^, γ = 10.2 under 365 nm exposure and PEB at 100°C for 3 min. DFT calculations revealed a six‐membered ring elimination mechanism, confirming that higher basicity and lower steric hindrance lead to lower reaction barriers and higher sensitivity. This work establishes a molecular‐level structure‐sensitivity relationship for base‐amplifying polymers, providing theoretical guidance for the rational design of high‐performance photoresists.

### 6.2 Copolymerization of sensitive monomers

As described in Section 2, the absorption efficiency of the PAG plays a decisive role in the sensitivity of the photoresist. One effective strategy to enhance this absorption efficiency is to incorporate photosensitive groups directly into the polymer structure via copolymerization.^[53]^ This copolymerization approach has been shown to effectively improve the uniformity of the photoresist film and its overall lithographic performance. As discussed above for EUV radiochemistry, resist performance under EUV exposure is strongly influenced by electron‐induced processes in addition to photon absorption itself. The incorporation of sensitive monomers may affect not only conventional chemical amplification behavior but also electron‐induced reaction pathways, local energy deposition, and ultimately the stochastic response under EUV exposure.

In parallel with the continued development of CARs, metal‐containing photoresists have also attracted increasing attention. They exhibit stronger EUV absorption, which helps reduce stochastic effects and defects. In addition, they are more capable of achieving higher ultimate resolution. On the other hand, relatively low sensitivity remains a major issue for some metal‐based photoresists.[^15^, ^102^] In contrast, CARs generally show limited EUV absorption and pronounced stochastic effects. To address this, incorporating metal elements into the structure of polymer‐based photoresists represents a promising strategy to balance the advantages and disadvantages of both systems.

**FIGURE 15:**
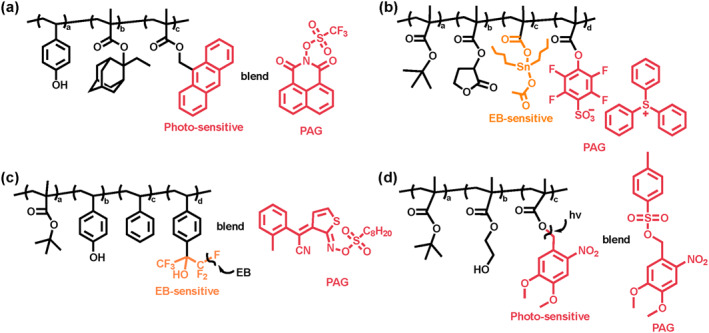
Strategies for enhancing photoresist sensitivity via copolymerization of photosensitive monomers. CARs sensitized by (a) copolymerized anthracene monomers; (b) copolymerized organotin‐containing monomers; (c) copolymerized hexafluoro‐2‐hydroxypropyl styrene; (d) dual‐sensitized CARs through the copolymerization of o‐nitrobenzyl monomers. CARs, chemically amplified resists.

Kim^[73]^ and colleagues designed chemically amplified terpolymers for negative‐tone development using *n*‐butyl acetate as an organic developer, incorporating 9‐anthracenylmethyl methacrylate (Figure 15a). With this negative‐tone imaging approach, they achieved well‐defined patterns with feature sizes as small as 3 μm using i‐line lithography, where lactone‐containing units provided the most pronounced solubility contrast and a sensitivity of 32 mJ/cm^2^. The use of organic developers avoids aqueous‐induced swelling and reduces LER, offering a promising pathway for high‐resolution EUV lithography without departing from conventional resist platforms.

Incorporating elements with high EUV absorption cross‐sections is a common strategy for enhancing the performance of photoresists used in advanced lithography nodes.[^103^, ^104^, ^105^] Yogesh and colleagues developed an organotin‐bearing tetrapolymer resist for EBL,^[106]^ achieving 33 nm resolution with high contrast (∼9.87) and good sensitivity (83 μC/cm^2^) (Figure 15b). The rational monomer design integrates acetoxydibutyltinmethacrylate as organotin sensitivity enhancer. The key advantages include thermal stability up to ∼195°C and low surface roughness (0.49 nm RMS).

A dual‐amplification strategy was demonstrated by Kang and coworkers in a high‐sensitivity resist based on a fluorinated copolymer containing hexafluoro‐2‐hydroxypropyl styrene (HFS) (Figure 15c).^[76]^ The remarkable performance—achieving 58 nm resolution with a low electron‐beam dose of 7 μC/cm^2^—originates from two synergistic effects. First, the high fluorine content enhances the absorption of high‐energy radiation. Second, and more critically, the radiation induces C–F bond cleavage, generating fluoride ions. These ions form hydrofluoric acid, which acts as a secondary potent catalyst to accelerate the deprotection reaction alongside the acid from the primary PAG.

Dual‐sensitization is a common design strategy for modern high‐sensitivity i‐line CARs.^[53]^ Zhu et al.^[107]^ employed a dual nonionic photoacid synergistic strategy by combining free PAG 4,5‐dimethoxy‐2‐nitrobenzyl p‐toluene sulfonate with polymer‐bound photoacid unit 4,5‐dimethoxy‐2‐nitrobenzyl methacrylate (MONMA) in P(MONMA‐HEMA‐TBMA) resist, achieving enhanced photosensitivity with 3 μm resolution at low exposure dose (857 mJ/cm^2^) while balancing acid diffusion and LER (Figure 15d).

### 6.3 Precision polymer architecture via controlled polymerization

The molecular weight distribution of a photoresist resin is paramount to achieving high‐fidelity nanopatterning. A broad distribution introduces significant variability in dissolution kinetics, which directly compromises pattern resolution and critically increases LER.^[108]^ Therefore, achieving a narrow monomodal molecular weight distribution through controlled polymerization techniques is not merely an optimization but a fundamental prerequisite for the development of next‐generation high‐performance photoresists.[^41^, ^109^] Therefore, to control the polydispersity of photoresist resins, researchers have widely adopted controlled/living polymerization techniques, such as reversible addition‐fragmentation chain‐transfer (RAFT) polymerization, atom transfer radical polymerization (ATRP), and living anionic polymerization.[^110^, ^111^]

**FIGURE 16:**
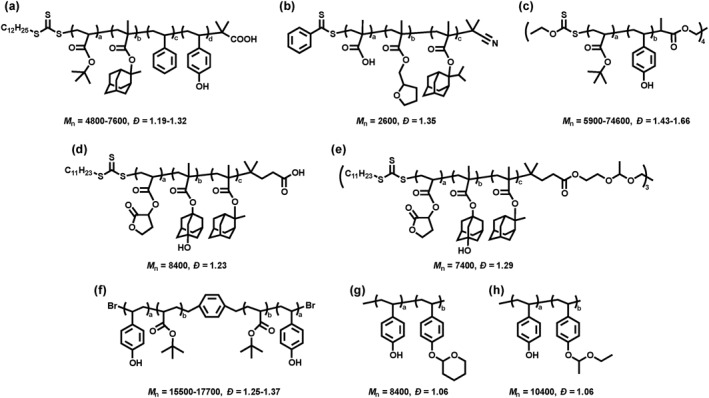
Examples of photoresist resin structures synthesized by controlled/living polymerization. Prepared by (a–e) RAFT polymerization; (f) atom transfer radical polymerization; and (g, h) living anionic polymerization.

Liu et al.^[112]^ used 2‐methyl‐2‐[(dodecylsulfanylthiocarbonyl)sulfanyl]propanoic acid (MDFC) as the RAFT agent to synthesize KrF photoresist polymers via RAFT polymerization with four (meth)acrylate monomers (styrene, 4‐acetoxystyrene, tert‐butyl acrylate, and 2‐methyl‐2‐adamantyl methacrylate) (Figure 16a). By varying the RAFT agent content, molecular weights were controlled in the range of 5710–10,110 g/mol with narrow polydispersity indices (*Đ*) of 1.19–1.32. The optimized photoresist exhibited 0.18 μm line/space resolution at an exposure energy of 16 mJ/cm^2^ under KrF lithography, demonstrating comparable performance to commercialized KrF photoresists.

Gao developed polymer‐bound PAG photoresists via RAFT polymerization, synthesizing methyl methacrylate copolymers (PTIMV) with controlled molecular weights and narrow *Đ* = 1.35 (Figure 16b).^[113]^ The RAFT‐controlled architecture ensures uniform PAG distribution along polymer chains, eliminating phase separation and surface segregation observed in conventional PAG‐blend systems. This molecular uniformity reduces acid diffusion length and surface roughness (*R*_q_ = 0.260 vs. 0.668 nm for blends), yielding lithographic patterns with improved morphology and lower LER despite slightly reduced sensitivity compared to blended counterparts.

Liu and coworkers synthesized star copolymers P(HS‐co‐TBA) via xanthate‐mediated RAFT polymerization with molecular weights (*M*_w_) of 8600–113,400 g/mol and low *Đ* between 1.43 and 1.52 (Figure 16c).^[114]^ As molecular weight decreased with increasing Star RAFT agent loading, photosensitivity improved (*E*_0_ from 4.8 to 3.2 mJ/cm^2^). The optimized star copolymer achieved 200 nm resolution at 12 mJ/cm^2^, outperforming linear analogs (250 nm).

The Kim group developed acid‐cleavable 3‐arm polymers (Figure 16e) via core‐first RAFT polymerization, where the star‐shaped architecture fundamentally differs from linear analogs (Figure 14d) in both degradation behavior and lithographic performance.^[115]^ Unlike linear polymers that rely solely on pendant group deprotection for solubility switching, the 3‐arm structure undergoes simultaneous molecular weight reduction to ≈30% of original *M*_n_ via acid‐catalyzed acetal cleavage at the core, generating three shorter chains. This dual‐mechanism approach dramatically enhances sensitivity: the 3‐arm terpolymer achieves ≈21% lower onset energy under DUV and ≈50% reduction under e‐beam compared to linear polymers, with pattern formation at ≈50% lower exposure energy. The star architecture enables sub‐100 nm e‐beam resolution unattainable with linear polymers, as the molecular weight reduction provides superior solubility modulation even at lower deprotection degrees, while maintaining comparable narrow polydispersity (*Đ* ≈ 1.29 vs. 1.23 for linear).

ATRP is difficult to apply to the synthesis of photoresist resins due to the involvement of metals during the polymerization process. However, recent studies have demonstrated that this issue can be resolved by employing ion exchange resins. Li synthesized ESCAP resin poly(AOST‐co‐TBA) via ATRP, achieving a weight‐average molecular weight (M_w_) of 22,100 g/mol and a low *Đ* of 1.25 (Figure 16f).^[116]^ To address the critical issue of metal ion contamination inherent to ATRP, they developed a purification process using ion exchange columns with Amberlite IRC747 and Amberlyst 15WET resins. The combined purification reduced the copper concentration from 34,700–1.12 ppb, with individual metal ions below 6 ppb and total metal ion concentration below 30 ppb, meeting the semiconductor industry requirement of <100 ppb for the first time in ATRP‐synthesized ESCAP resins.

Anionic polymerization is well‐known for yielding polymers with narrow polydispersity, and thus holds great promise for the preparation of photoresist resins.^[117]^ Ryu and coworkers synthesized poly(4‐hydroxystyrene)‐based copolymers via anionic polymerization, achieving molecular weights (*M*_n_ = 7000–11,300 g/mol) and extremely narrow polydispersity (*Đ* = 1.05) (Figure 16g,h).^[118]^ The well‐defined architecture ensures uniform dissolution and minimal LER. Final copolymers exhibit high thermal stability (T_g_ up to 143°C) and low UV absorption at 248 nm (0.179/μm).

**FIGURE 17:**
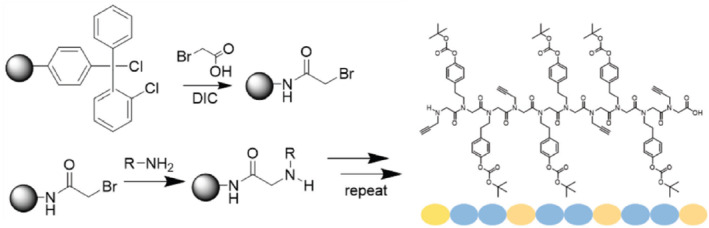
Solid phase‐supported synthesis of polypeptoids photoresist using tyramine and propargylamine as primary amines. Reproduced with permission.^[120]^ Copyright 2025, ACS Publications.

Furthermore, it has been demonstrated that the monomer sequence within the polymer chain also plays a crucial role in determining the overall lithographic performance.^[119]^ Ober and coworkers introduced polypeptoids as a new platform for CARs via solid‐phase submonomer synthesis (Figure 17).[^120^, ^121^] Initial studies using 10‐mer sequences with *t*‐Boc‐protected tyramine and benzylamine demonstrated that sequence influences DUV patterning, with hydrophobic end groups improving imaging performance. Extending this approach to EUV lithography, they incorporated propargylamine as a hydrophobic unit alongside 4‐ethyl phenol. EUV exposures revealed that homogeneous distribution of hydrophobic units significantly outperformed block‐like sequences, achieving 70 nm pitch (35 nm half‐pitch) patterns with a dose‐to‐clear of approximately 10 mJ/cm^2^, establishing sequence control as a critical parameter for EUV resist performance.

### 6.4 Photosensitized chemically amplified resists

**FIGURE 18:**
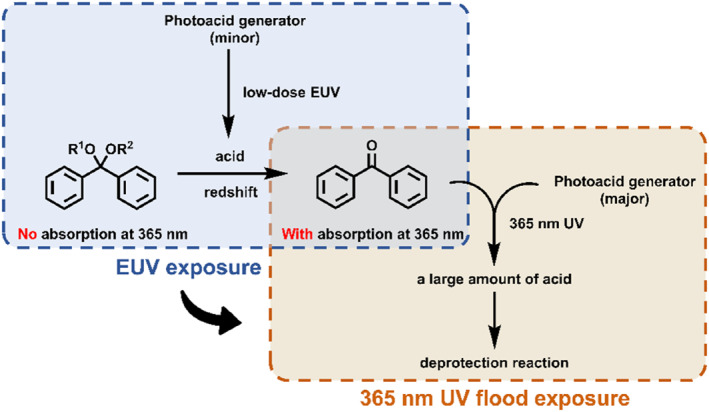
Schematic diagram illustrating the working principle of the photosensitized chemically amplified resists.

Given the relatively low energy of EUV light, researchers have proposed a two‐step exposure sensitization strategy— photosensitized CARs (PSCARs). PSCARs represent an innovative strategy to overcome the inherent RLS trade‐off in EUV lithography. The PSCAR system incorporates a photosensitizer precursor into conventional CARs formulations.[^122^, ^123^, ^124^, ^125^, ^126^] Upon EUV exposure, this precursor is activated to liberate an active photosensitizer, which subsequently enhances photoacid generation during a subsequent mid‐UV (365 nm) flood exposure (Figure 18). This dual‐exposure process selectively increases acid concentration in EUV‐exposed regions, thereby improving sensitivity while maintaining high resolution. Advanced iterations such as PSCAR 2.0 further integrate photo‐decomposable base quenchers that can be simultaneously photosensitized during flood exposure, enhancing acid image contrast through selective quencher decomposition.^[127]^ Despite demonstrating promising performance with 16 nm dense line‐space patterns, the multi‐component nature of PSCAR systems presents practical challenges requiring continued optimization for high‐throughput manufacturing applications.

## 7 CONCLUSION AND OUTLOOK

Recent decades have witnessed significant advances in patterning materials for photolithography, a progression necessitated by the demand for smaller feature sizes and the use of lower‐wavelength light sources. The development of photoresists for advanced lithography nodes faces several key challenges and trends: (1) for advanced technology nodes, resist sensitivity is insufficient, largely due to the low photon utilization efficiency inherent to advanced lithography; (2) incomplete mechanistic understanding: the fundamental working mechanism of EUV photoresists is not yet fully understood; (3) approaching the fundamental limits of the RLS trade‐off: As CD continue to shrink, the impact of chemical stochastic effects—such as random acid diffusion and discrete reaction events—becomes increasingly pronounced. This exacerbates the fundamental trade‐off between resolution, LER, and sensitivity, making it extraordinarily difficult to simultaneously optimize all three parameters.

A synergistic approach that integrates computational and experimental chemistry is a crucial strategy for elucidating a comprehensive EUV mechanism. In terms of material synthesis, for PAGs, key development goals will be driven by enhanced structure‐property relationship studies, focusing on: (1) achieving high absorption cross‐sections at exposure wavelengths; (2) maximizing quantum yields for acid generation; (3) minimizing acid diffusion to improve resolution; (4) reducing or eliminating unwanted side reactions. For photoresist resins, future research directions will include: (1) achieving precise control over polymer dispersity (i.e., synthesizing polymers with narrow molecular weight distributions); (2) designing highly labile protecting groups with exceptional sensitivity to acids or bases; (3) exploring novel amplification and sensitization strategies, and elucidating the interplay between these mechanisms (such as main‐chain scission, self‐immolation, or self‐amplification) and the ultimate lithographic performance; (4) developing materials with high etch resistance and selectivity for pattern transfer. Building on the reviewed advancements, we are now exploring the aforementioned strategies in our laboratory.

## CONFLICT OF INTEREST STATEMENT

The authors declare no conflicts of interest.

## Data Availability

Data sharing not applicable to this article as no datasets were generated or analyzed during the current study.
